# Dietary Probiotics Modulate Oxidative Stress, Metabolic Status, and Immune-Related Gene Expression in Nile Tilapia (*Oreochromis niloticus*) Exposed to Malathion

**DOI:** 10.3390/vetsci13050441

**Published:** 2026-04-30

**Authors:** Abdullah A. A. Alghamdi

**Affiliations:** Department of Biology, Faculty of Science, Al-Baha University, Al-Baha 65779, Saudi Arabia; aaa.alghamdi@bu.edu.sa

**Keywords:** organophosphate exposure, dietary intervention, aquaculture toxicology, antioxidant defense, immunomodulation, recovery physiology

## Abstract

Malathion, a widely used organophosphate pesticide, frequently contaminates aquatic ecosystems and poses considerable toxic risks to non-target organisms, including fish. The present study provides an integrated evaluation of the protective effects of dietary probiotics against malathion-associated oxidative, metabolic, and immune-related disturbances in Nile tilapia at the biochemical and molecular levels. After determining the 96 h LC_50_ of malathion, fish were exposed to a sublethal concentration for 7 days followed by a 14-day recovery period while receiving either a basal or probiotic-supplemented diet. Malathion exposure increased cumulative mortality, induced behavioral stress, and caused metabolic and hepatorenal disturbances characterized by elevated serum glucose and cholesterol, altered serum protein fractions, increased alanine and aspartate aminotransferase activities, and elevated creatinine and uric acid levels. Oxidative stress was evidenced by increased serum malondialdehyde and transcriptional suppression of antioxidant-related genes (*sod-2* and *cat*) in the liver, spleen, and intestine. Malathion also triggered immune dysregulation through the upregulation of pro-inflammatory cytokine genes (*il-1β* and *tnf-α*) and suppression of regulatory cytokines (*tgf-β* and *il-10*). Probiotic supplementation during recovery significantly reduced mortality, restored metabolic and hepatorenal biomarkers, attenuated oxidative damage, and enhanced antioxidant capacity at both the biochemical and transcriptional levels. Moreover, probiotic-supplemented fish exhibited controlled pro-inflammatory signaling accompanied by the pronounced activation of regulatory cytokines, indicating balanced immune modulation. Collectively, dietary probiotics effectively mitigate malathion-induced toxicity by improving antioxidant defense, immune regulation, and physiological resilience, highlighting their potential as functional dietary additives for sustainable aquaculture in Nile tilapia.

## Simple Summary

This study investigated whether probiotic supplementation to the diet of Nile tilapia could mitigate the toxic effect of malathion and improve the overall health status of the fish. The results showed that exposure to malathion caused severe stress, organ damage, and immune system dysfunction. Conversely, probiotic supplementation reduced mortality, restored metabolic and hepatorenal function, enhanced antioxidant defense, and improved immune regulation and physiological resilience. Collectively, probiotic supplementation effectively mitigates malathion-induced toxicity in Nile tilapia through the coordinated modulation of oxidative stress, metabolic function, and immune responses.

## 1. Introduction

For decades, pesticides have been extensively applied in agriculture to enhance crop productivity and control disease vectors [1]. Among these chemicals, organophosphorus compounds are widely used in agriculture, industry, and public health applications [2]. Malathion is a non-systemic, broad-spectrum organophosphate insecticide that has been extensively applied worldwide [3], largely due to its relatively short environmental persistence and high selectivity toward target pests [4,5]. However, its intensive use, particularly near aquatic environments, has resulted in the frequent contamination of freshwater and marine ecosystems, thereby posing serious risks to non-target organisms such as fish [6,7].

Malathion inhibits acetylcholinesterase (AChE), preventing the central and peripheral nervous systems from hydrolyzing the neurotransmitter acetylcholine [8]. Fish are adversely affected by malathion, even at low concentrations [9]. Sublethal concentrations of malathion induce oxidative stress and tissue damage in *Oreochromis niloticus* because of alterations in biochemical and hematological features [6,10]. Accumulating evidence clearly indicates that malathion exposure disrupts redox homeostasis in fish, primarily through excessive reactive oxygen species (ROS) generation and the subsequent impairment of antioxidant defense systems [11]. Pesticide-induced oxidative stress signifies a familiar mechanism underlying toxic outcomes in aquatic organisms [12]. Additionally, malathion exposure leads to hepatorenal toxicity in fish, categorized by the malfunction of liver enzymes and altered renal biochemical markers, indicative of interrupted nitrogenous metabolism [13]. Organophosphate pesticides demonstrated the interruption of immune homeostasis in fish through oxidative stress as well as an alteration in key innate immune responses, relating redox imbalance to immune dysregulation under toxic conditions [12,14].

Pesticides cause damage to the tissues and vital organs of fish and disrupt their biological and behavioral functions, leading to increased mortality rates [15]. The production of protein through sustainable aquaculture has become an appropriate approach to addressing the projected global demand. In this regard, tilapia is the second most farmed fish species in more than 100 countries [16]. To meet the growing aquatic food demand, aquaculture methods should be developed to enhance productivity [17]. Accordingly, nutritional strategies that can strengthen physiological resilience before or during toxicant exposure are of increasing interest in modern aquaculture [16,18,19]. Studies have demonstrated that the use of probiotics as functional feed additives enhances the physiological resilience of fish by improving immune responsiveness and antioxidant defense, suggesting that they may also serve as a preventative dietary approach against pesticide-induced stress [16,18,19].

The term probiotics refers to live microorganisms that, when taken in adequate quantities, confer health benefits on the host [20]. In recent years, probiotics have increasingly been used in aquaculture to control diseases without reliance on antibiotics or chemical therapeutics [16]. The utilization of mixed-species probiotics in tilapia aquaculture represents a promising nutritional strategy for sustainable production. The dietary supplementation of tilapia with probiotics has been shown to boost growth performance, nutrient utilization, and disease resistance [21,22,23,24]. It is believed that probiotics enhance the function of the epithelial barrier, improve adhesion to intestinal cells, inhibit pathogens by occupying adhesion sites, and regulate immune function [25]. In addition to these established benefits, earlier studies suggest that probiotics may exert protective effects not only against pathogenic challenges but also against chemical stressors through the modulation of oxidative stress responses and immune-related gene expression, thereby enhancing physiological resilience in fish [18,26]. This raises the possibility that probiotic supplementation may function not only as a supportive treatment after stress exposure but also as a preventative nutritional measure capable of reducing the severity of pesticide-induced physiological disruption [18,26].

While previous research has examined the toxicological impacts of malathion on fish physiology and health, comprehensive studies investigating how dietary probiotics modulate oxidative stress, metabolic disturbance, and immune-related gene expression under malathion stress in Nile tilapia remain lacking. In particular, the preventative potential of probiotics against pesticide-induced toxicity has not been sufficiently clarified in an integrated biochemical and transcriptional framework. Addressing this gap is important for determining whether probiotic supplementation can serve as a practical and sustainable strategy to improve resilience and support recovery in aquaculture systems facing chemical contamination. Therefore, this study evaluated whether dietary probiotic supplementation could modulate biochemical responses, oxidative stress-related alterations, immune-related gene expression, and recovery in Nile tilapia under malathion exposure.

## 2. Materials and Methods

### 2.1. Fish Source

Apparently healthy Nile tilapia housed in 500 L holding fiberglass tanks within a recirculating aquaculture system in the departmental aquatic laboratory were used in this study. Water quality parameters were carefully maintained: total ammonia at 0.1 ± 0.02 mg/L, pH at 7.2 ± 0.2, temperature at 25.1 ± 0.5 °C, and dissolved oxygen at 6.2 ± 0.5 mg/L.

### 2.2. Diets and Chemicals Used in the Experiment

Briefly, Nile tilapia was subjected to two distinct dietary treatments. The first treatment comprised a commercial diet suitable for the species, containing 30% crude protein, set as the basal diet. The second consisted of an enriched diet, formulated by supplementing the basal feed with a 3 g/kg diet of a commercially available AquaStar^®^ Growout probiotic blend (Biomin GmbH, Getzersdorf, Austria) mix of *Bacillus*, *Enterococcus*, *Lactobacillus* and *Pediococcus* probiotic blend (minimum 1 × 10^9^/g). The probiotic blend was initially homogenized in water and subsequently applied evenly to the feed via spraying. Fresh diets were prepared daily to ensure high probiotic viability.

The insecticide used in this study was a commercially available emulsifiable concentrate of malathion containing 57% active ingredient (PT AMCO International, Jakarta, Indonesia).

### 2.3. Determination 96h LC_50_ of Malathion

Acute toxicity was evaluated to determine the lethal concentration 50% (LC_50_) of malathion in Nile tilapia, as described earlier by [27]. Briefly, a total of 270 Nile tilapia were randomly divided into eight experimental groups, each comprising 30 fish (*n* = 10/replicate/100 L water) in glass aquaria. The fish were exposed to eight different concentrations of malathion, 4, 2, 1, 0.5, 0.25, 0.125, 0.06, and 0.03 ppm, for 96 h without diet supplement to avoid the undesirable effect of excreta and feed [28]. An alternative group of 30 fish was also simultaneously retained in dechlorinated water (0 ppm) as a control. All concentrations, including the control, were tested in triplicate. Continuous aeration was maintained throughout the trial to ensure adequate dissolved oxygen levels.

Daily water exchange (30%) was performed throughout the exposure period to maintain optimal water quality, and malathion concentrations were reconstituted to ensure consistent exposure levels. Mortality rates (%) were recorded at 24, 48, 72, and 96 h following the onset of malathion exposure to evaluate the acute toxic effects on Nile tilapia. Following the determination of LC_50_, half of the concentration was used for the subsequent experiments. The sublethal concentration used in this experiment 0.5 × LC_50_ was selected to induce measurable physiological stress without causing excessive mortality, which allows for the evaluation of the protective effects of dietary probiotics under toxic conditions.

### 2.4. Experimental Setup

**Figure 1:**
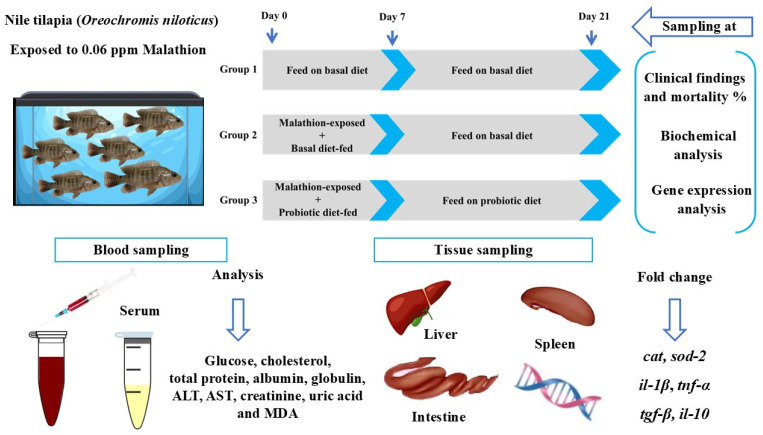
A schematic representation of the experimental design, sample collection, and methodology. The experiment comprised three groups organized into two sets, each with three replicates. The first set, designated for sampling, included 30 fish per replicate, while the second set, used for recording mortality, contained 10 fish per replicate.

Following the determination of LC_50_, 270 healthy juvenile Nile tilapia, each with an average initial body weight between 35 and 40 g (2.5 months old), were randomly distributed into 9 flow-through tanks, each containing 30 fish/300 L water. Figure 1 depicts the experiment setup and methodology.

Fish tanks were organized into three experimental groups, with three replicates per group:

- Group 1 (control): Fish were continuously fed a basal diet for 21 days without exposure to malathion.
- Group 2 (malathion-exposed/basal diet-fed): Fish were exposed to malathion and fed a basal diet for 7 days, followed by a 14-day recovery period on a basal diet only.
- Group 3 (malathion-exposed/probiotic diet-fed): Fish were exposed to malathion and fed a basal diet supplemented with a probiotic product for 7 days, followed by a 14-day recovery period on the same probiotic-supplemented diet only.

Notably, the 14-day recovery period was chosen based on preliminary trials which indicate that tilapia typically show restored biochemical and immune parameters within two weeks post-stress.

Three additional groups, mirroring the original experimental design, were established specifically for monitoring mortality. Each experimental group consisted of a total of 30 Nile tilapia, with 10 fish/100 L water allocated per replicate tank (*n* = 90). These groups followed the same treatment protocols, control, malathion exposure with basal diet feed, and malathion exposure with enriched diet feed, but were dedicated to recording mortality outcomes throughout the exposure and recovery periods.

During the experimental period, a fixed feeding ration was implemented to maintain consistent nutritional conditions across all groups. Fish were fed once daily. A controlled photo-period of 12 h light and 12 h dark was also kept. Water quality parameters were routinely monitored to ensure optimal conditions for fish health and growth. Approximately 30% of the aquarium water was replaced daily to maintain water quality, and malathion concentrations were reconstituted to ensure consistent exposure levels.

### 2.5. Collection of Blood Samples

At the beginning of the experiment (zero day), and at the end of both the malathion exposure period (7 days) and the subsequent recovery phase (14 days), six fish from each tank were randomly selected and anesthetized using clove oil [29], resulting in a total of 18 fish per treatment group at each sampling point. Immediately after anesthesia, blood was drawn from the caudal vessels using sterile syringes and transferred into clean Eppendorf tubes. The samples were centrifuged at 1500× *g* for 15 min at 4 °C to separate serum, which was carefully collected and amassed at −20 °C for later biochemical evaluations.

### 2.6. Tissue Sampling

Immediately following blood collection at the same sampling time points, fish were euthanized using an overdose of clove oil. Intestine, liver, and spleen tissues from the three fish in each group were preserved in RNAlater™ (Thermo Fisher Scientific, Waltham, MA, USA), refrigerated all night, and subsequently stored at −80 °C to maintain RNA integrity.

### 2.7. Analysis of Serum Biochemical Parameters

For the biochemical evaluation, the serum obtained from three fish within each replicate was pooled, yielding six composite samples per treatment group (*n* = 6). Pooling was performed to obtain sufficient volume for accurate biochemical analyses. The pooled sample was treated as one biological replicate representing the experimental unit (tank). This approach ensured adequate sample volume while minimizing variability related to individual sampling and handling. Biochemical indices were measured using commercial assay kits (Bio-Diagnostics, Worcestershire, UK) following the manufacturer’s instructions. The evaluated parameters comprised glucose, cholesterol, total protein, albumin, globulin, alanine aminotransferase (ALT), aspartate aminotransferase (AST), uric acid, creatinine, and malondialdehyde (MDA). Computations were performed using a T80 UV/VIS spectrophotometer (PG Instruments Limited, Leicestershire, UK).

Glucose levels were determined according to [30], while cholesterol was measured following the methods of [31,32]. Total protein and albumin concentrations were analyzed utilizing the procedures defined by [33] and [34], respectively. Globulin values were estimated as outlined by [35]. The activities of AST and ALT were assessed using the method of [36]. Creatinine and uric acid concentrations were quantified following [37] and [38], respectively. MDA levels were measured based on the protocol of [39].

### 2.8. RNA Extraction, cDNA Synthesis and RT-qPCR

#### 2.8.1. RNA Extraction and Preparation of Complementary DNA

Total RNA was isolated from tissue specimens utilizing the RNeasy Mini Kit (Qiagen GmbH, Hilden, Germany), strictly following the manufacturer’s procedure. RNA concentration and purity were determined by a spectrophotometer with an Implen NanoPhotometer^®^ spectrophotometer (Implen GmbH, Munich, Germany).

For first-strand complementary DNA (cDNA) synthesis, 1 μg of total RNA (2.1 ng/μL ± 0.2 SD) was reverse-transcribed using the RevertAid First Strand cDNA Synthesis Kit (Thermo Scientific, Waltham, MA, USA) according to the manufacturer’s guidelines. The resulting cDNA products were stored at −20 °C until further use in real-time quantitative polymerase chain reaction (RT-qPCR) assays.

#### 2.8.2. Target Genes and Primer Design

To assess the differential expression of selected genes, RT-qPCR was performed targeting *il-1β*, *tnf-α*, *tgf-β*, *il-10*, *sod-2*, and *cat*. Gene-specific primers were employed for each target, with the sequences detailed in Table 1. For the normalization of gene expression data, the *β-actin* gene of Nile tilapia was used as the internal reference control.

#### 2.8.3. Relative Quantification of Gene Expression

Quantitative real-time PCR was conducted by applying the HERAPLUS SYBR Green qPCR Kit (Willowfort Ltd., Birmingham, UK) on the Mx3005P^®^ real-time QPCR detection system (Agilent Technologies, Santa Clara, CA, USA), following the manufacturer’s protocol. Each 20 μL reaction contained 10 μL of 2× SYBR Green master mix, 1 μL of each gene-specific primer (10 pmol/μL), 2 μL of cDNA template, and sterile, nuclease-free PCR-grade water to adjust the final volume. No-template controls were included to monitor potential contamination.

Regarding the thermal cycling conditions for *il-1β*, *tgf-β*, *il-10*, *sod-2*, *cat* and the reference gene (*β-actin*), a two-step protocol was used: initial denaturation at 95 °C for 3 min, followed by 40 cycles of 95 °C for 10 s and 60 °C for 1 min, with annealing and extension combined. For *tnf-α*, the protocol consisted of an initial denaturation at 95 °C for 3 min, tracked by 40 cycles of 95 °C for 30 s, 58 °C for 30 s, and 72 °C for 30 s.

Fluorescence data were collected during the extension phase. Primer efficiencies, determined via standard curve analysis, ranged between 90% and 100%. Amplification specificity was confirmed by melt curve analysis (65 °C to 95 °C, incrementing by 0.5 °C every 0.05 s), which showed single peak profiles. Cycle threshold (Ct) values were determined, and relative gene expression levels were estimated employing the 2^−ΔΔCt^ system as defined by [47].

### 2.9. Statistical Analysis

All data are presented as the mean ± standard error of the mean (SEM). Prior to conducting an analysis of variance (ANOVA), the assumptions of normality and homogeneity of variance were tested. Both assumptions were satisfied, validating the use of an ANOVA for the dataset. The statistical significance of the control and exposed groups was evaluated using one- and two-way ANOVA. Differences were considered statistically significant at *p* < 0.05. Post hoc comparisons among treatment means were accomplished using Tukey’s multiple-range test. All statistical analyses were carried out using GraphPad Prism software, version 8.0.1 (GraphPad Software, San Diego, CA, USA).

## 3. Results

### 3.1. LC_50_-96h

**Figure 2:**
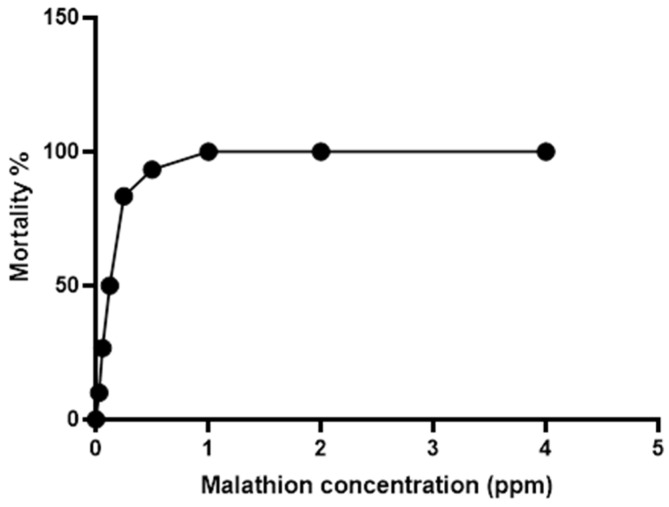
Concentration–mortality curve for Nile tilapia (*n* = 30/concentration in triplicates) exposed to malathion for 96 h, with median lethal concentration (LC_50_) determination.

Mortality trends in fish at 96h of malathion exposure are shown in Figure 2. The findings indicated that 0.125 ppm represented LC_50_, while 0.06 ppm was identified as half LC_50_, making it the preferred concentration for subsequent experiments because it reliably induced measurable physiological stress without excessive mortality, allowing us to evaluate recovery dynamics.

### 3.2. Observations of Health Distress in Nile Tilapia

Nile tilapia exposed to malathion for 7 days and fed with a basal diet exhibited pronounced respiratory distress, including open-mouth breathing, rapid opercular movement, frequent surfacing, and erratic swimming behavior. External signs included excessive mucus secretion covering the skin, congested gills with increased mucus production, fin rot, sloughing of scales, and visible hemorrhages in the brain. The internal organs were notably congested and enlarged. In contrast, fish exposed to malathion and fed with a probiotic-enriched diet showed markedly reduced signs of health distress. After a recovery period of 14 days, both fish groups appeared normal, with only mild fin sloughing.

### 3.3. Mortality Trends

**Figure 3:**
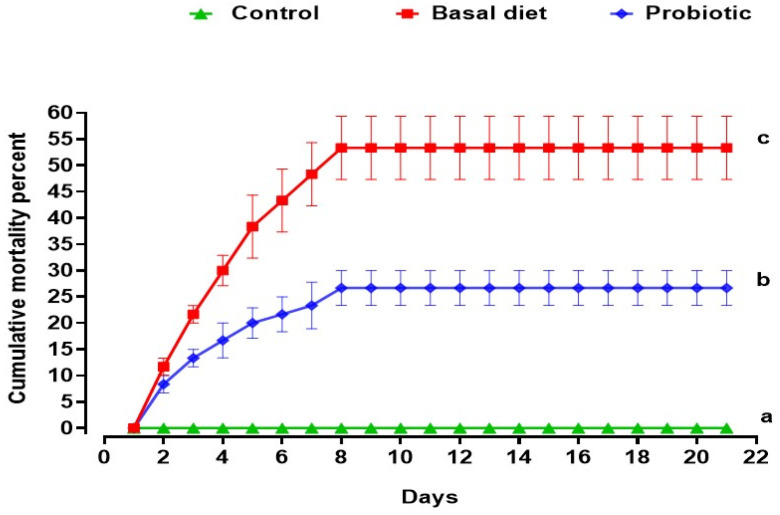
The cumulative mortality percent over 21 days in Nile tilapia during 0.06 ppm malathion exposure and recovery. Fish were fed either a basal diet or a probiotic-supplemented diet throughout the experimental period. The control group received the basal diet without malathion exposure. Mortality was recorded at baseline, after 7 days of malathion exposure, and following a 14-day recovery period. Statistically significant differences among groups were determined by a one-way ANOVA (*p* < 0.05) and are indicated by distinct lowercase letters.

The cumulative mortality rates over the 21-day experimental period (Figure 3) showed significant differences among the groups. The control group exhibited 0% mortality throughout this study, confirming the reliability of the experimental conditions. Mortality in both malathion-exposed groups began on the second day of exposure and ceased one day after the exposure ended, following a similar temporal pattern. However, the cumulative mortality differed significantly between the groups: fish exposed to malathion and fed the basal diet had a mortality rate of 53.3% ± 6.0, whereas those fed the probiotic-enriched diet showed a substantially lower mortality rate of 26.7% ± 3.3.

### 3.4. Serum Biochemical Parameters

At the beginning of the experiment (day 0), all experimental groups exhibited comparable values across all assessed parameters, with no statistically significant differences observed among them.

**Figure 4:**
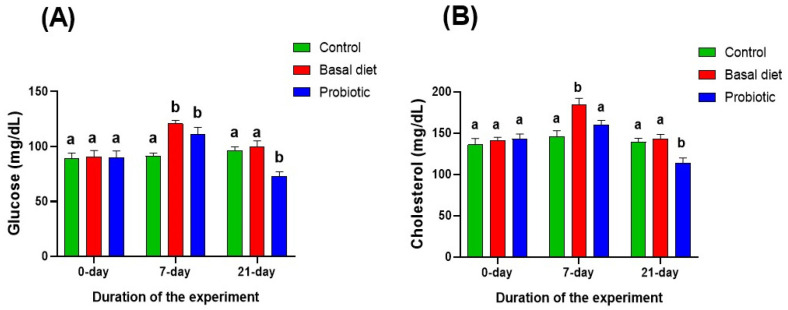
Changes in serum glucose (**A**) and cholesterol (**B**) concentrations in Nile tilapia at the start of the experiment, after 7 days of exposure to 0.06 ppm malathion, and following the 14-day recovery period. Fish were provided with either a basal diet or a probiotic-supplemented diet throughout the experimental period. The control group received the basal diet without malathion exposure. Data are expressed as the mean ± SEM (*n* = 6). Statistically significant differences among groups at each time point were determined by a two-way ANOVA (*p* < 0.05) and are denoted by distinct lowercase letters.

Following 7 days of malathion exposure, serum glucose levels (Figure 4A) were significantly elevated in both exposed groups relative to the control. In contrast, a significant increase in serum cholesterol (Figure 4B) was observed only in fish receiving the basal diet. After a 14-day recovery period, fish fed the basal diet exhibited the normalization of both glucose and cholesterol concentrations to control values. Notably, probiotic-supplemented fish showed a significant decrease in both glucose and cholesterol levels compared with the basal diet and control groups.

**Figure 5:**
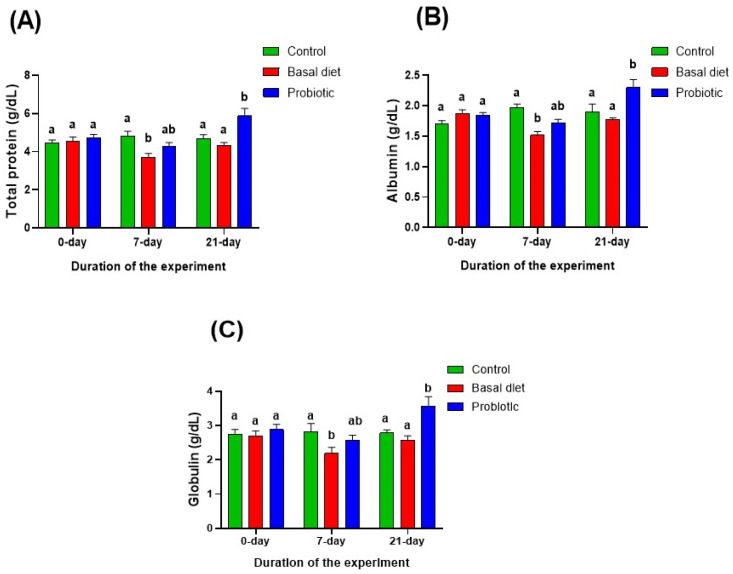
Changes in serum total protein (**A**), albumin (**B**) and globulin (**C**) concentrations in Nile tilapia at the start of the experiment, after 7 days of exposure to 0.06 ppm malathion, and following the 14-day recovery period. Fish were provided with either a basal diet or a probiotic-supplemented diet throughout the experimental period. The control group received the basal diet without malathion exposure. Data are expressed as the mean ± SEM (*n* = 6). Statistically significant differences among groups at each time point were determined by a two-way ANOVA (*p* < 0.05) and are denoted by distinct lowercase letters.

After 7 days of malathion exposure, serum total protein, albumin, and globulin levels (Figure 5A–C) were significantly reduced in fish fed the basal diet. In contrast, fish receiving the probiotic-enriched diet exhibited a non-significant decline in these parameters compared with both the control and basal diet groups. Following the recovery period, these parameters in the basal diet group normalized to control levels. Interestingly, probiotic feeding resulted in higher protein fraction levels compared with both the control and basal diet groups.

**Figure 6:**
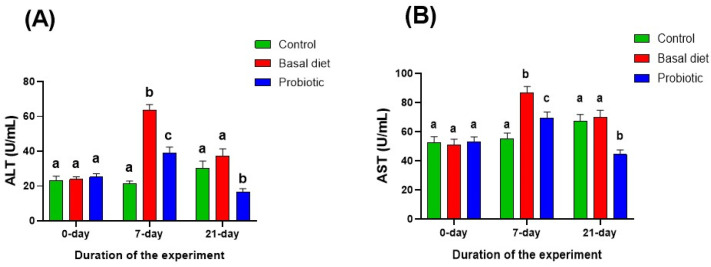
Changes in serum alanine aminotransferase (ALT) (**A**) and aspartate aminotransferase (AST) (**B**) in Nile tilapia at the start of the experiment, after 7 days of exposure to 0.06 ppm malathion, and following the 14-day recovery period. Fish were provided with either a basal diet or a probiotic-supplemented diet throughout the experimental period. The control group received the basal diet without malathion exposure. Data are expressed as the mean ± SEM (*n* = 6). Statistically significant differences among groups at each time point were determined by a two-way ANOVA (*p* < 0.05) and are denoted by distinct lowercase letters.

Following malathion exposure, serum ALT and AST activities (Figure 6A,B) were significantly elevated in both experimental groups. These elevations were normalized after recovery on the basal diet. In contrast, probiotic supplementation during recovery resulted in significantly reduced ALT and AST activities compared with both the control and basal diet groups.

**Figure 7:**
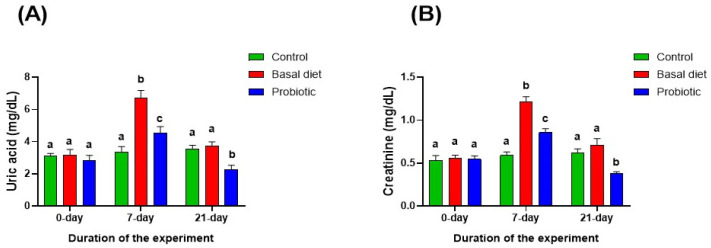
Changes in serum uric acid (**A**) and creatinine (**B**) levels in Nile tilapia at the start of the experiment, after 7 days of exposure to 0.06 ppm malathion, and following the 14-day recovery period. Fish were provided with either a basal diet or a probiotic-supplemented diet throughout the experimental period. The control group received the basal diet without malathion exposure. Data are expressed as the mean ± SEM (*n* = 6). Statistically significant differences among groups at each time point were determined by a two-way ANOVA (*p* < 0.05) and are denoted by distinct lowercase letters.

Malathion exposure significantly increased serum uric acid and creatinine (Figure 7A,B) in both exposed groups, particularly in the basal diet group. These alterations normalized after recovery on the basal diet, whereas probiotic supplementation resulted in significantly lower levels than those observed in the control and basal diet groups.

**Figure 8:**
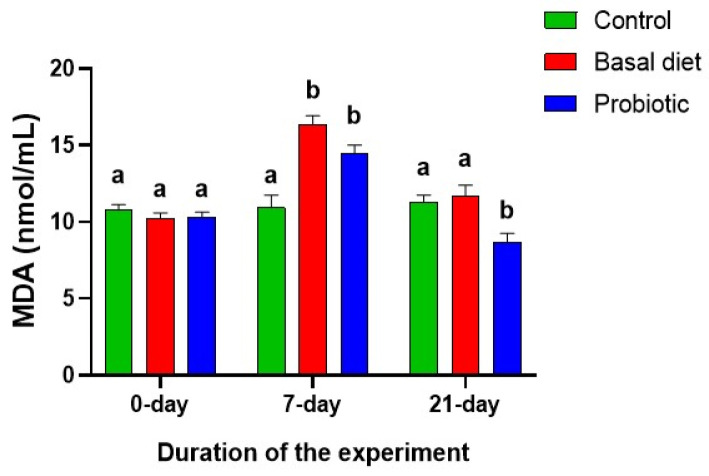
Changes in serum malondialdehyde (MDA) levels in Nile tilapia at the start of the experiment, after 7 days of exposure to 0.06 ppm malathion, and following the 14-day recovery period. Fish were provided with either a basal diet or a probiotic-supplemented diet throughout the experimental period. The control group received the basal diet without malathion exposure. Data are expressed as the mean ± SEM (*n* = 6). Statistically significant differences among groups at each time point were determined by a two-way ANOVA (*p* < 0.05) and are denoted by distinct lowercase letters.

Malathion exposure significantly elevated serum MDA levels (Figure 8) in both exposed groups; however, these levels normalized following recovery on the basal diet, while probiotic supplementation resulted in significantly lower MDA levels than those in the control and basal diet groups.

### 3.5. Gene Expression Analysis

At the start of the experiment (day 0), no significant differences were detected in the expression of any of the analyzed genes among the experimental groups.

**Figure 9:**
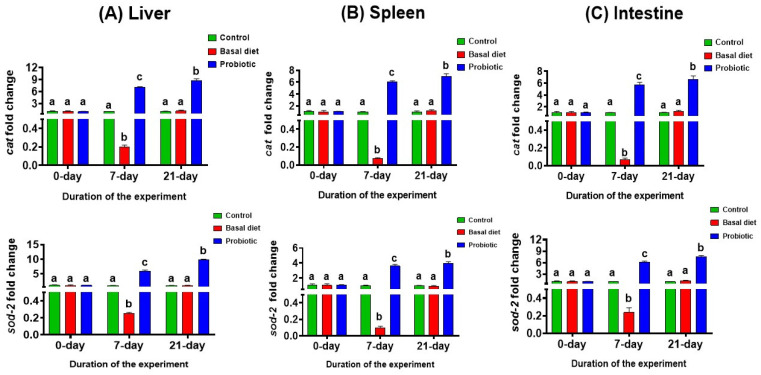
The expression levels of antioxidant genes *cat* and *sod-2* in the liver (**A**), spleen (**B**) and intestinal (**C**) tissues in Nile tilapia at the start of the experiment, after 7 days of exposure to 0.06 ppm malathion, and following the 14-day recovery period. Fish were provided with either a basal diet or a probiotic-supplemented diet throughout the experimental period. The control group received the basal diet without malathion exposure. Data are expressed as the mean ± SEM (*n* = 3). Statistically significant differences among groups at each time point were determined by a two-way ANOVA (*p* < 0.05) and are denoted by distinct lowercase letters.

Following 7 days of malathion exposure, the antioxidant-related genes *cat* and *sod-2* were significantly downregulated in the liver, spleen, and intestine of Nile tilapia fed the basal diet compared with the control group. In contrast, fish receiving the probiotic-enriched diet showed a significant upregulation of both genes across all examined tissues relative to the control. After the recovery period, gene expression in the basal diet group returned to levels comparable to those of the control fish. Notably, probiotic supplementation during recovery induced a significant upregulation of *cat* and *sod-2*, exceeding the expression levels observed in both the control and basal diet groups (Figure 9A–C).

**Figure 10:**
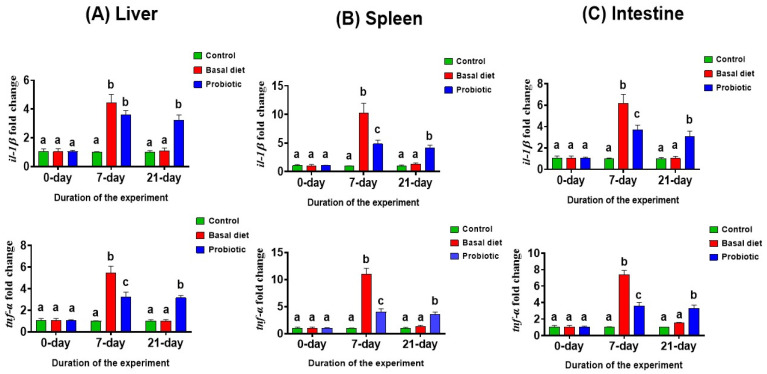
The expression levels of pro-inflammatory cytokine genes *il-1β* and *tnf-α* in the liver (**A**), spleen (**B**) and intestinal (**C**) tissues in Nile tilapia at the start of the experiment, after 7 days of exposure to 0.06 ppm malathion, and following the 14-day recovery period. Fish were provided with either a basal diet or a probiotic-supplemented diet throughout the experimental period. The control group received the basal diet without malathion exposure. Data are expressed as the mean ± SEM (*n* = 3). Statistically significant differences among groups at each time point were determined by a two-way ANOVA (*p* < 0.05) and are denoted by distinct lowercase letters.

Following 7 days of malathion exposure, the pro-inflammatory genes *il-1β* and *tnf-α* were significantly upregulated in the liver, spleen, and intestine of both exposed groups, particularly in the basal diet group. After the recovery period, gene expression in the basal diet group returned to levels comparable to those of the control fish. Notably, probiotic supplementation during recovery induced a significant upregulation of *il-1β* and *tnf-α*, exceeding the expression levels observed in both the control and basal diet groups (Figure 10A–C).

**Figure 11:**
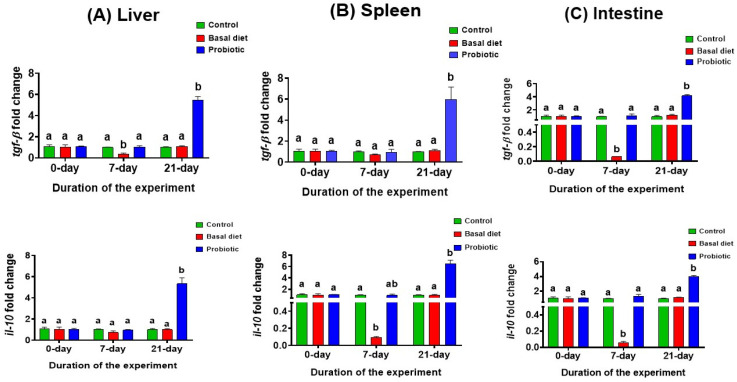
The expression levels of anti-inflammatory cytokine genes *tgf-β* and *il-10* in the liver (**A**), spleen (**B**) and intestinal (**C**) tissues in Nile tilapia at the start of the experiment, after 7 days of exposure to 0.06 ppm malathion, and following the 14-day recovery period. Fish were provided with either a basal diet or a probiotic-supplemented diet throughout the experimental period. The control group received the basal diet without malathion exposure. Data are expressed as the mean ± SEM (*n* = 3). Statistically significant differences among groups at each time point were determined by a two-way ANOVA (*p* < 0.05) and are denoted by distinct lowercase letters.

In the basal diet group, following 7 days of malathion exposure, the anti-inflammatory genes *tgf-β* in the liver and *il-10* in the spleen were significantly downregulated compared to the control, and both genes were significantly downregulated in the intestine. In contrast, fish fed the probiotic-enriched diet maintained expression levels comparable to the control across all examined tissues. After the recovery period, expression in the basal diet group returned to control levels. Notably, probiotic supplementation during recovery induced a significant upregulation of *tgf-β* and *il-10*, surpassing the levels observed in both the control and basal diet groups (Figure 11A–C).

## 4. Discussion

The present study determined the 96 h LC_50_ of malathion for Nile tilapia to be 0.125 ppm. This value differs from that reported by Pathiratne and George [48], who recorded a 96 h LC_50_ of 2.2 ppm, and from the findings of Al-Ghanim [49], who reported a 96 h LC_50_ of 1.06 mg L^−1^ for Nile tilapia. Such variation among reported LC_50_ values may be attributed to differences in experimental conditions. These factors include fish size and age, water quality parameters and exposure duration and the formulation of pesticides, as well as the absorption, accumulation, biotransformation and excretion of pesticides [50].

Taken together, the present findings suggest that malathion exposure in Nile tilapia induced a biologically integrated stress syndrome in which oxidative imbalance, metabolic disruption, hepatorenal dysfunction, and immune dysregulation were mechanistically interconnected rather than representing isolated toxic manifestations [11,12,13,14,51,52]. Within this framework, the probiotic effect is more convincingly interpreted as a stabilizing influence on physiological homeostasis than as a simple reversal of individual biomarkers [18,26,53,54,55,56].

This study demonstrates that dietary probiotic supplementation plays a pivotal role in modulating the physiological, oxidative, and immunological disturbances induced by malathion exposure in Nile tilapia. Consistent with previous reports describing malathion as a potent inducer of oxidative stress and immune dysregulation in fish [14,53], malathion exposure in the present study resulted in marked alterations in serum biochemical indices, antioxidant defenses, and inflammatory gene expression. Rather than merely returning selected endpoints to baseline, probiotic supplementation appeared to redirect the overall recovery trajectory towards a more regulated physiological state [53,55,56]. This interpretation is important because it implies that probiotic supplementation influenced recovery across several systems simultaneously, which is a more meaningful biological outcome than improvement in any single variable alone [54,55].

Malathion exposure induced clear toxic effects in Nile tilapia, as evidenced by dose-dependent mortality, characteristic behavioral and clinical signs of distress, and increased cumulative mortality during the exposure period. Such responses are consistent with the well-documented toxicity of organophosphate pesticides in fish, which primarily act through the inhibition of acetylcholinesterase activity, leading to neuromuscular dysfunction and respiratory impairment [48,57]. The determination of LC_50_ values and the observed temporal pattern of mortality confirm the suitability of the selected sublethal concentration for inducing physiological stress while minimizing excessive acute lethality, thereby allowing for a reliable assessment of recovery-related physiological and molecular responses. The reduced mortality and attenuation of clinical distress in probiotic-supplemented fish suggest that the dietary treatment improved resilience during toxic challenge, possibly by lowering the physiological cost of oxidative and inflammatory stress [18,26,53].

Oxidative stress is a well-recognized consequence of organophosphate exposure in aquatic organisms, primarily resulting from excessive ROS generation and the subsequent impairment of antioxidant defenses [11,12]. In the present study, the oxidative component of malathion toxicity appears to extend beyond lipid peroxidation alone and is likely to contribute to broader cellular and tissue dysfunction [11,12,14]. Similar increases in MDA levels have widely been reported in teleosts exposed to malathion and other organophosphate pesticides and are considered hallmark indicators of pesticide-induced oxidative stress [14,57]. The attenuation of lipid peroxidation in probiotic-fed fish therefore suggests the improved control of ROS-driven membrane damage, which may have limited the secondary amplification of cellular injury during recovery [18,53,56]. This reduction suggests an improved oxidative balance and enhanced capacity to counteract ROS-mediated cellular damage, consistent with previous studies demonstrating the antioxidant supporting potential of dietary probiotics in fish under toxic or environmental stress conditions [18,53].

At the molecular level, the attenuation of malathion-induced oxidative stress was supported by the consistent modulation of antioxidant-related gene expression across all examined tissues. Fish exposed to malathion and fed the basal diet exhibited a significant downregulation of *sod-2* and *cat*, indicating a generalized suppression of antioxidant defenses under toxic stress, which is consistent with the excessive oxidative burden associated with pesticide biotransformation [11,57]. This transcriptional pattern is biologically meaningful because superoxide dismutase and catalase function sequentially in ROS detoxification, with the former converting superoxide radicals into hydrogen peroxide and the latter eliminating hydrogen peroxide before it can promote further oxidative injury [11,12]. Through this sequential scavenging pathway, the probiotic-associated upregulation of *sod-2* and *cat* may help limit radical propagation, reduce oxidative injury to cellular macromolecules, and promote the restoration of redox homeostasis in pesticide-stressed tissues [11,12]. Although glutathione peroxidase was not quantified in the present study, the *sod-2*/*cat* response may reasonably be viewed as part of a broader antioxidant enzyme network that constrains ROS escalation in pesticide-stressed tissues [11,12,58]. Such a response suggests that probiotics strengthened endogenous antioxidant preparedness rather than merely reducing the visible biochemical consequences of oxidative stress [26,54,58]. In contrast, probiotic supplementation markedly enhanced the expression of these antioxidant genes during both the exposure and recovery phases, reflecting an improved antioxidant capacity and more effective control of oxidative damage [18,53]. Similar protective patterns across tissues suggest that probiotics promote a coordinated, systemic activation of antioxidant defense mechanisms rather than organ-specific effects. This sustained upregulation of *sod-2* and *cat* likely underlies the improved redox homeostasis and enhanced recovery capacity observed in probiotic-treated fish, reinforcing the role of probiotics in mitigating pesticide-induced oxidative stress [26,54]. The biological plausibility of this protective effect is strengthened by the known functional properties of the probiotic genera included in the preparation, namely *Bacillus*, *Enterococcus*, *Lactobacillus*, and *Pediococcus*, which are widely associated with improved gut health, mucosal defense, and immune regulation in tilapia [25,43,59]. These genera have been associated with improved gut barrier function, the competitive exclusion of undesirable microbes, enhanced nutrient utilization, and the modulation of mucosal and systemic immune responses, all of which may contribute to improved tolerance to toxic stress in fish [25,43,59,60]. It is therefore plausible to hypothesize that part of the probiotic benefit observed here may have arisen within the gut–pesticide axis, where dietary microbes could influence an early interface between toxic exposure, mucosal immunity, nutrient absorption, and systemic stress signaling [25,43,59,61]. This possibility deserves emphasis because intestinal dysfunction may exacerbate oxidative stress and inflammatory signaling and may contribute to broader physiological instability in fish [25,62,63]. Recent work in Nile tilapia supports this view by showing that probiotic supplementation can mitigate pesticide-related intestinal injury and immune impairment, while other studies in tilapia have shown probiotic-associated improvement in gut health, mucosal architecture, and barrier-related functions [25,64,65]. In this context, the present antioxidant and cytokine responses may reflect not only systemic protection but also improved intestinal resilience that may have helped limit the wider propagation of toxic stress within the organism [25,26,53].

The disruption of carbohydrate and lipid metabolism is a well-documented consequence of organophosphate exposure in fish [51,66]. Consistent with a previous study [6], malathion exposure induced a significant elevation in serum glucose levels during the exposure phase. Such elevations in glucose have been attributed to the increased catecholamine and cortisol secretion, which stimulate gluconeogenesis and glycogenolysis to meet intensified energy demands under toxic stress [11,57]. From a physiological perspective, this response reflects a shift towards emergency energy mobilization under toxic challenge [66]. Following the recovery period, glucose levels in malathion-exposed fish fed with the basal diet reverted to values comparable to the control group, thus indicating the partial normalization of carbohydrate metabolism once the toxic pressure was removed. The more pronounced improvement in probiotic-supplemented fish suggests that probiotic administration may have supported more effective metabolic readjustment during recovery rather than merely permitting a passive return to baseline [53,55]. Similar hypoglycemic effects of dietary probiotics have been reported in Nile tilapia and other fish species, where probiotics were shown to enhance metabolic efficiency, improve hepatic function, and modulate glucose homeostasis under stress conditions [18,53].

A comparable pattern was observed in cholesterol dynamics. Malathion exposure led to a marked increase in serum cholesterol, particularly in fish fed the basal diet, which is in complete agreement with earlier studies linking organophosphate toxicity to impaired lipid metabolism and altered hepatic lipid processing [11,14]. This pattern supports the interpretation that probiotic supplementation contributed to the improved regulation of lipid metabolism during recovery, potentially through effects on hepatic function and the mitigation of metabolic stress [53,55]. This finding aligns well with previous studies, demonstrating that probiotics can improve lipid profiles by enhancing bile acid metabolism, regulating lipid synthesis, and supporting liver integrity in fish exposed to environmental or chemical stressors [18,26]. Collectively, these findings suggest that probiotic supplementation plays an active role in restoring metabolic homeostasis following malathion exposure, not merely by reversing stress-induced hyperglycemia and hypercholesterolemia but by promoting a more efficient regulation of carbohydrate and lipid metabolism during the recovery phase.

Serum protein fractions and hepatic transaminases are widely recognized as sensitive indicators of liver functions integrity and metabolic capacity in fish exposed to environmental toxicants [52]. In the present study, malathion exposure induced a marked reduction in serum total protein, albumin, and globulin levels during the exposure phase, concomitant with pronounced elevations in ALT and AST activities. These alterations are indicative of impaired hepatic synthetic function and increased hepatocellular membrane permeability, thereby facilitating the leakage of intracellular enzymes [11,57]. Similar biochemical manifestations of hepatic dysfunction, including reduced circulating protein fractions and elevated transaminase activities, have been consistently reported in fish exposed to malathion and other organophosphate pesticides, reflecting hepatocellular injury and the disruption of normal liver metabolism [11,48].

A limited capacity for spontaneous hepatic recovery was observed in malathion-exposed fish fed with the basal diet following the recovery period. In contrast, probiotic supplementation markedly enhanced hepatic recovery, as evidenced by significant increases in serum total protein, albumin, and globulin levels together with pronounced reductions in ALT and AST activities. This pattern supports the interpretation that probiotic supplementation contributed to the improved regulation of lipid metabolism during recovery, potentially through effects on hepatic function, nutrient utilization, and the alleviation of metabolic stress [55,67,68]. Evidence from Nile tilapia studies further supports that probiotic administration can normalize stress-related biochemical disturbances, including the restoration of serum protein fractions and attenuation of transaminase activities under toxic conditions [53,55]. Overall, these findings demonstrate that probiotic supplementation mitigates malathion-induced hepatic dysfunction not only by limiting hepatocellular damage, as reflected by reduced ALT and AST activities, but also by promoting the recovery and enhancement of hepatic protein synthesis.

Renal function biomarkers, such as serum uric acid and creatinine, are commonly employed as sensitive indicators of kidney integrity and excretory capacity in fish exposed to environmental contaminants [52]. In the present study, malathion exposure resulted in a pronounced elevation in serum creatinine levels. Nitrogenous waste indicators such as uric acid also increased, during the exposure phase, indicating impaired renal function and the reduced efficiency of nitrogenous waste excretion. Similar malathion-associated increases in renal biomarkers, particularly creatinine and other nitrogenous waste indicators, have been reported in fish and are widely interpreted as biochemical signs of nephrotoxicity following organophosphate exposure [13,69]. Comparable elevations in renal biomarkers have been reported in fish exposed to malathion and other pesticides, where oxidative stress, vascular congestion, and tubular degeneration are considered key mechanisms underlying pesticide-induced renal dysfunction [52].

A partial normalization of uric acid and creatinine levels was observed in malathion-exposed fish following a period of recovery after the removal of the toxicant. In contrast, probiotic supplementation was associated with reduced serum uric acid and creatinine levels during recovery, suggesting a partial improvement in renal biochemical status relative to the malathion-exposed basal diet group. This improvement is consistent with the possibility that probiotics reduced the systemic burden of oxidative and metabolic stress affecting the kidney, thereby supporting improved renal functional status during recovery [55,56]. Further evidence suggests that dietary probiotics can ameliorate stress-related renal disturbances through the mitigation of oxidative damage, stabilization of cellular membranes, and improvement in systemic metabolic balance under toxic conditions [55,56]. Overall, these findings suggest that probiotic supplementation plays a protective role in preserving renal function during recovery from malathion exposure, thereby complementing its beneficial effects on hepatic and metabolic homeostasis.

Pro-inflammatory cytokines such as IL-1β and TNF-α play central roles in the initiation and amplification of inflammatory responses in fish following exposure to environmental stressors and xenobiotics [70]. Pesticides, including malathion, are well documented to activate these inflammatory pathways through oxidative stress-mediated signaling and tissue injury [70].

Malathion exposure markedly upregulated hepatic, splenic, and intestinal *il-1β* and *tnf-α* expression after 7 days in fish fed the basal diet, reflecting a robust inflammatory response to pesticide-induced stress. Similar elevations in pro-inflammatory cytokines have been consistently reported in teleosts exposed to malathion and other organophosphates and are considered characteristic indicators of toxicant-induced inflammation [13,57].

The probiotic-related cytokine profile in the present study does not suggest indiscriminate suppression of inflammatory signaling but rather a more controlled pattern of immune responsiveness [53,59]. This distinction is important because recovery from toxic stress would be expected to require the preservation of immune competence rather than the complete silencing of pro-inflammatory pathways [53,70]. Probiotic supplementation was associated with the attenuation of *il-1β* and *tnf-α* induction during the exposure phase relative to the malathion-exposed basal diet group, while during recovery, it was associated with the sustained upregulation of both pro-inflammatory and regulatory cytokine transcripts, a pattern more consistent with immune modulation than with excessive or dysregulated inflammation [53,70,71]. Notably, probiotic supplementation significantly attenuated the magnitude of *il-1β* and *tnf-α* induction during the exposure phase across all examined tissues. Although cytokine expression in the probiotic-treated group remained higher than that in the control fish, it was consistently lower than that observed in the malathion-exposed basal diet group, indicating a partial but biologically meaningful modulation of the inflammatory response. This pattern suggests that probiotics reduced the inflammatory burden without completely suppressing host immune activation, a phenomenon widely described as immune modulation rather than immune inhibition [26].

Taken together, the attenuation of *il-1β* and *tnf-α* induction during exposure, followed by sustained elevation in both pro-inflammatory and regulatory cytokine transcripts during recovery in probiotic-supplemented fish, is more consistent with controlled immune modulation than with unresolved pathological inflammation, particularly because this pattern coincided with improved antioxidant status, hepatorenal function, and survival in the present study [53,70,71]. Because the present molecular dataset is limited to mRNA abundance, this pattern should be interpreted as evidence of controlled transcriptional responsiveness rather than direct proof of cytokine protein production, sustained inflammatory activity, or definitive immune priming.

Such persistent but controlled elevation in pro-inflammatory cytokines has been previously reported in probiotic-fed fish and is more consistent with regulated immune responsiveness than with pathological inflammation [26]. Recently, Hou et al. demonstrated that probiotic supplementation sustained moderate cytokine expression while improving overall health and stress resistance [19], supporting the concept that probiotics fine-tune immune responsiveness instead of abolishing inflammatory signaling.

Regulatory anti-inflammatory cytokines, comprising *tgf-β* and *il-10*, are significant mediators of immune homeostasis, functioning to limit excessive inflammation and promote resolution and tissue repair [70,71]. The disruption of these regulatory pathways is commonly associated with uncontrolled inflammatory responses following toxicant exposure in fish [14]. In the present study, malathion exposure caused a reduction in *tgf-β* and *il-10* expression in the liver, spleen, and intestine after 7 days in fish fed the basal diet, indicating the suppression of anti-inflammatory regulatory mechanisms during the exposure phase. A similar downregulation of regulatory cytokines under pesticide-induced stress has been reported in teleosts and is often linked to immune dysregulation and heightened susceptibility to tissue damage [11]. In contrast, probiotic-supplemented fish maintained hepatic, splenic, and intestinal *tgf-β* and *il-10* expression at levels comparable to the control group during the exposure phase, suggesting the preservation of regulatory immune balance despite ongoing malathion challenge. This finding aligns well with previous studies demonstrating the capacity of probiotics to stabilize immune regulation and prevent excessive inflammatory escalation under stress conditions [18,53].

During recovery, the return of *tgf-β* and *il-10* expression to control levels in the malathion-exposed basal diet group contrasted with the sustained upregulation of both regulatory cytokines across the examined tissues in probiotic-supplemented fish on day 21. This pattern is more consistent with the active immunoregulatory support of recovery than with passive resolution after toxicant withdrawal, particularly because it coincided with improved antioxidant status, hepatorenal function, and survival in the present study [53,70,71].

The broader implications of these findings are directly relevant to current efforts to improve aquaculture sustainability in areas exposed to agricultural runoff and chemical contamination [7]. A feed-based probiotic intervention is practically attractive because it can be incorporated into routine husbandry and may provide a biologically realistic means of improving fish robustness where the complete control of waterborne pollutants is difficult to achieve [26,72]. For species such as Nile tilapia, which contribute substantially to affordable animal protein production in many regions, strategies that lessen the physiological cost of pesticide exposure may also have wider implications for production stability and food security [72,73]. There may also be an ecological dimension, since interventions that reduce the biological burden of contaminant exposure in cultured fish could indirectly lessen the impact of chemical stress in aquaculture-relevant aquatic environments [7,60].

Taken together with the antioxidant, metabolic, hepatic, and renal findings, the present results suggest that dietary probiotics may serve as practical functional tools for mitigating pesticide-induced physiological stress in aquaculture [53,55]. At the same time, the mechanistic interpretation should remain appropriately cautious, since the present study did not include histopathology, protein-level or functional validation, or a direct assessment of gut microbiota composition.

## 5. Conclusions

The present study suggests that dietary probiotic supplementation may help mitigate malathion-associated physiological stress in Nile tilapia by improving oxidative, metabolic, and immune-related responses. Probiotic treatment was associated with reduced lipid peroxidation, enhanced antioxidant-related responses, and reduced mortality during exposure and recovery. The observed cytokine transcription patterns were more consistent with regulated immune modulation than with dysregulated inflammation; however, these findings should be interpreted cautiously because the molecular data were limited to mRNA expression. Overall, the results support the potential value of probiotics as functional dietary tools for improving fish resilience under organophosphate-related stress in aquaculture systems.

## Figures and Tables

**Table 1:** Primers used for real-time PCR.

Primer Name	Primer Sequences (5′–3′)	Accession Number	Reference
*β-actin* F	CAGGATGCAGAAGGAGATCACA	KJ126772.1	[40]
*β-actin* R	CGATCCAGACGGAGTATTTACG		
*il-1β* F	TGCACTGTCACTGACAGCCAA	DQ061114	[41]
*il-1β* R	ATGTTCAGGTGCACTATGCGG		
*tnf-α* F	GGAAGCAGCTCCACTCTGATGA	JF957373.1	[42]
*tnf-α* R	CACAGCGTGTCTCCTTCGTTCA		
*tgf-β* F	GTTTGAACTTCGGCGGTACTG	NM_001311325.1	[43]
*tgf-β* R	TCCTGCTCATAGTCCCAGAGA		
*il-10* F	CTCAGATGGAGAGCAGAGGTC	KP645180.1	[44]
*il-10* R	CTTGATTTGGGTCAGCAGGT		
*cat* F	TCCTGGAGCCTCAGCCAT	JF801726	[45]
*cat* R	ACAGTTATCACACAGGTGCATCTTT		
*sod-2* F	CTCCAGCCTGCCCTCAA	JF801727.1	[46]
*sod-2* R	TCCAGAAGATGGTGTGGTTAATGTG		

## Data Availability

All data generated or analyzed during this study are included in this article, while raw data is available from the author on request.
